# Exposure to Poly- and Perfluoroalkyl Substances During Pregnancy and Asthma in Childhood: A Systematic Review

**DOI:** 10.7759/cureus.73568

**Published:** 2024-11-13

**Authors:** Maria-Melanthia Aeraki, Dimitra Metallinou, Athina Diamanti, Vasiliki E Georgakopoulou, Antigoni Sarantaki

**Affiliations:** 1 Department of Midwifery, University of West Attica, Athens, GRC; 2 Department of Pathophysiology/Pulmonology, Laiko General Hospital, Athens, GRC

**Keywords:** asthma, childhood, pfas, prenatal exposure, respiratory outcomes

## Abstract

Asthma is a chronic respiratory disease that has been increasing in prevalence among children globally. While genetic factors contribute to asthma risk, there are growing concerns about environmental exposures, such as poly- and perfluoroalkyl substances (PFAS), which may disrupt respiratory and immune development in the prenatal period. This systematic review aimed to assess the association between prenatal PFAS exposure and the development of asthma in childhood, integrating findings from epidemiological studies. A comprehensive literature search was conducted on Scopus, PubMed, and Google Scholar using predefined keywords. The review included prospective cohort studies examining prenatal PFAS exposure and asthma outcomes in children. The quality of the studies was assessed using the Newcastle-Ottawa Scale (NOS).

A total of 13 studies were included in the final analysis. The studies involved diverse countries/populations with over 21,000 mother-child pairs. Although some studies reported associations between specific PFAS compounds and asthma, most found no significant relationship between prenatal PFAS exposure and doctor-diagnosed asthma. The findings were inconsistent, with null or mixed results across studies. Despite mechanistic evidence suggesting that PFAS could contribute to asthma development, epidemiological data do not consistently support a link between prenatal exposure and childhood asthma. Further research with standardized exposure measurements and larger cohort studies is needed to clarify these associations.

## Introduction and background

Asthma, a chronic respiratory disease with considerable prevalence among children globally, has witnessed a concerning rise in incidence over recent decades, representing a growing public health challenge [1]. While genetic predispositions are central to asthma susceptibility, there is growing evidence that environmental exposures, particularly during critical developmental windows such as the prenatal period, may modulate immune and respiratory health, shaping disease risk in early life and beyond [2-4].

Among environmental factors of growing concern are poly- and perfluoroalkyl substances (PFAS), a diverse class of synthetic chemicals with unique water- and grease-resistant properties that make them ubiquitous in both industrial applications and consumer products [5]. PFAS are a class of synthetic chemicals widely used in a variety of consumer products, including non-stick cookware, water-resistant fabrics, and food packaging, thanks to their ability to repel water and grease. This versatility has led to PFAS becoming common in everyday items [5]. However, PFAS compounds are highly persistent, bioaccumulative, and pervasive in human tissues, detectable in the placenta, umbilical cord blood, and breast milk. This widespread presence has raised significant concerns regarding their potential to impact fetal development and contribute to adverse health outcomes with lifelong repercussions [6-8].

Recent research has highlighted possible associations between prenatal PFAS exposure and various deleterious health effects, including immunotoxicity, endocrine disruption, and respiratory impairment [9-11]. Given that the fetal immune and respiratory systems are especially sensitive to environmental perturbations, prenatal exposure to PFAS may disrupt immune homeostasis and respiratory function, potentially heightening asthma risk in childhood [12]. Nonetheless, the evidence for this relationship remains fragmented, with studies offering divergent findings; while some identify a positive correlation, others report null associations [13-15]. This inconsistency highlights the need for a comprehensive, methodologically robust synthesis of the available data to better elucidate these associations.

This systematic review seeks to critically assess and integrate the current body of epidemiological literature regarding prenatal PFAS exposure and the risk of developing asthma in childhood. Through this synthesis, the review aims to clarify the potential role of these persistent chemicals in asthma pathogenesis, advancing our understanding of a field with profound public health implications.

## Review

Materials and methods

Search Strategy

We conducted a comprehensive literature search on Scopus, PubMed, and Google Scholar databases to identify studies that examine the association between prenatal exposure to environmental PFAS, and the development of asthma in childhood. The search terms used included combinations of (“PFAS” OR “perfluoroalkyl” OR “polyfluoroalkyl” OR “chemical exposure”) AND (“childhood asthma” OR “respiratory outcomes” OR “asthma risk” OR “immunological outcomes”). The systematic review protocol was registered with the International Prospective Register of Systematic Reviews (PROSPERO) to ensure transparency and adherence to systematic review standards with ID number 606235.

Inclusion and Exclusion Criteria

This systematic review adhered to the Preferred Reporting Items for Systematic Reviews and Meta-Analyses (PRISMA) guidelines [16]. The inclusion criteria were as follows - a) study designs: prospective cohort, cross-sectional, retrospective cohort studies and clinical trials published in peer-reviewed journals; b) population: pregnant women with documented exposure to PFAS and subsequent asthma-related outcomes in their children; c) outcomes of interest: childhood asthma incidence; and c) language: only studies published in English were included.

We excluded meta-analyses, review articles, case reports, editorials, and studies not focused on prenatal exposures to PFAS relevant to childhood asthma.

Exposure and Outcome Definition

The primary exposure of interest was maternal exposure to PFAS during pregnancy, with levels assessed through biological samples (e.g., maternal blood, cord blood) reflecting prenatal chemical load. The primary outcome measure was childhood asthma diagnosis.

PRISMA Process

**Figure 1:**
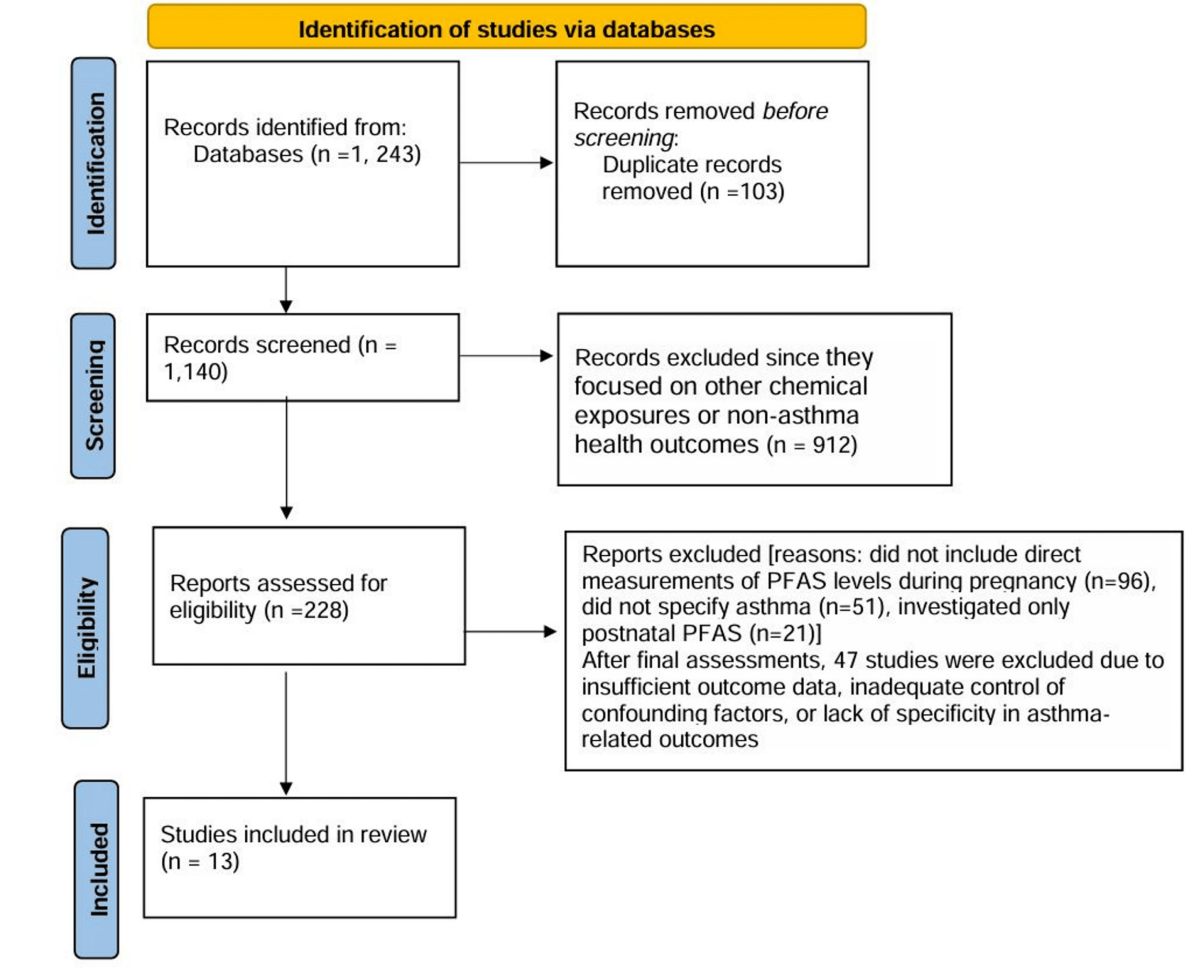
PRISMA flowchart depicting the study selection process PRISMA: Preferred Reporting Items for Systematic Reviews and Meta-Analyses

The study selection process is displayed in Figure 1. 

Identification and Screening

Our initial search across Scopus, PubMed, and Google Scholar yielded 1,243 articles. After removing 103 duplicate entries, 1,140 unique studies remained. These were screened based on predefined inclusion and exclusion criteria, resulting in the exclusion of 912 studies that were not relevant, such as those focused on other chemical exposures or non-asthma health outcomes. This left 228 studies for full-text review.

Eligibility and Inclusion

A full-text review of these 228 articles was conducted to verify that each study measured prenatal PFAS exposure and reported specific childhood asthma. After this evaluation, 168 studies were excluded for the following reasons: i) 96 studies did not include direct measurements of PFAS levels during pregnancy; ii) 51 studies did not specify asthma or respiratory-related outcomes and iii) 21 studies investigated only postnatal PFAS exposure.

This left 60 studies that met the inclusion criteria. After final assessments, 47 studies were excluded due to insufficient outcome data, inadequate control of confounding factors, or lack of specificity in asthma-related outcomes. Ultimately, 13 studies were selected for data synthesis.

Quality Assessment

The methodological quality of the included studies was assessed using the Newcastle-Ottawa Scale (NOS) [17], evaluating selection, comparability, and outcome assessment. Studies were scored, and only those meeting a minimum quality threshold were included in the final analysis to ensure the reliability and validity of the findings.

Data Extraction

Two reviewers independently extracted relevant data from each included study, such as authors, publication year, country, study design, sample size, level of PFAS in maternal or cord blood, and measured outcomes (asthma diagnosis). Any discrepancies in data extraction were resolved via consensus or through consultation with a third reviewer.

Results

This systematic review analyzed 13 studies from diverse countries/populations [13-15,18-27], including Norway, France, Denmark, the USA, China, Spain, Ukraine, Japan, and the Faroe Islands, with a combined sample size of over 21,000 mother-child pairs. All studies were prospective cohort designs, examining the impact of prenatal PFAS exposure on childhood asthma and respiratory health outcomes. PFAS exposure was measured primarily based on maternal or cord blood during pregnancy, and asthma-related outcomes were followed up in children from early childhood to adolescence. Table 1 summarizes the characteristics of the included studies.

**Table 1:** Characteristics of the included studies COPSAC2010: Copenhagen Prospective Studies on Asthma in Childhood 2010; ECA: Environment and Childhood Asthma study; HR: hazard ratio; ISAAC: International Study of Asthma and Allergies in Childhood; MMR: measles, mumps, and rubella (vaccine); NOS: Newcastle-Ottawa Scale; OR: odds ratio; PFAS: per- and polyfluoroalkyl substances; PFHxS: perfluorohexane sulfonate; PFNA: perfluorononanoate; PFOA: perfluorooctanoate; PFOS: perfluorooctane sulfonate; PFUnDA: perfluoroundecanoic acid; SEPAGES - SEPAGES cohort (no expanded abbreviation provided); VDAART: Vitamin D Antenatal Asthma Reduction Trial; BraMat: BraMat cohort; MoBa: Norwegian Mother, Father and Child Cohort Study; FDR: false discovery rate; IgE: immunoglobulin E; PCBs: polychlorinated biphenyls; DDE: dichlorodiphenyldichloroethylene

Authors	Year	Country	Type of study	Study sample	Measured parameter	Outcomes	Key findings	NOS
Granum et al. [13]	2013	Norway	Prospective cohort study	99 participants from BraMat cohort	PFAS levels in maternal blood	Infectious diseases, allergy-related diseases, vaccine antibody levels	Asthma-related outcomes: doctor-diagnosed asthma: no significant association between prenatal PFAS exposure and the occurrence of doctor-diagnosed asthma in children by age three. Other allergy indicators: similarly, no link was found between PFAS exposure and other allergy indicators, including eczema, wheezing, or atopic eczema	5/9
Impinen et al. [14]	2018	Norway	Prospective birth cohort study	641 participants from the ECA study	PFAS levels in cord blood	Respiratory tract infections, asthma, allergy-related diseases	Asthma-related outcomes: no significant associations between prenatal PFAS exposure and asthma diagnosis at two or 10 years of age. No significant associations were found between PFAS levels and other allergy-related outcomes such as allergic rhinitis, eczema, or allergic sensitization	6/9
Philippat et al. [18]	2023	France	Prospective birth cohort study	433 mother-child pairs from the SEPAGES cohort	PFAS levels in maternal blood, lung function parameters (oscillometry)	Respiratory symptoms (wheezing, asthma, bronchitis, bronchiolitis), lung function measures	Asthma outcomes: no significant association was found between prenatal PFAS exposure and doctor-diagnosed asthma up to age three	7/9
Beck et al. [15]	2019	Denmark	Prospective birth cohort study	981 mother-child pairs from the Odense Child Cohort	PFAS levels in maternal serum; asthma and wheeze symptoms via ISAAC-based questionnaire	Wheezing symptoms, asthma prevalence (self-reported, doctor-diagnosed), sex-specific associations	Asthma and wheezing: 18.6% reported wheezing, and 7.1% reported asthma, with 4.5% having doctor-diagnosed asthma. PFAS and asthma outcomes: no significant association between PFAS and doctor-diagnosed asthma or wheezing. Significant association for self-reported asthma with PFNA exposure (OR: 1.84). Sex-specific findings: higher odds of self-reported asthma in boys and stronger associations with doctor-diagnosed asthma in girls, particularly for PFHxS exposure	7/9
Begum et al. [19]	2024	USA	Prospective cohort study within a randomized controlled trial	459 mother-child pairs from the VDAART cohort	PFAS levels in maternal serum (3rd trimester); clinician-diagnosed asthma up to age eight	Childhood asthma incidence	Asthma risk with PFAS exposure: prenatal PFAS exposure was not significantly associated with an increased risk of asthma. Adjusted HRs for individual PFAS compounds were elevated in some cases, but all confidence intervals (CIs) included the null. Mixed PFAS exposure effects: the PFAS mixture showed a non-significant trend (HR 1.17 per quartile increase), not reaching statistical significance. Conclusion: no consistent relationship between prenatal PFAS exposure and asthma incidence in early childhood	6/9
Sevelsted et al. [20]	2023	Denmark	Prospective birth cohort study (COPSAC2010)	738 mother-child pairs	PFOS and PFOA levels in maternal plasma; childhood asthma phenotypes (atopic vs. non-atopic)	Asthma phenotypes (atopic and non-atopic), sensitization, lung function, epigenetics	Non-atopic asthma: higher prenatal PFOS and PFOA exposure linked to increased risk of non-atopic asthma (OR: 1.20 for PFOS; OR: 2.81 for PFOA). No association with atopic asthma: no link was observed between prenatal PFAS exposure and atopic asthma, lung function, or atopic dermatitis. Allergic sensitization: prenatal PFOS exposure was associated with a reduced risk of sensitization to inhalant allergens. Mechanistic insights: no significant associations with immune response markers or epigenetic changes, suggesting other pathways for PFAS effects on asthma phenotypes	7/9
Timmermann et al. [21]	2017	Faroe Islands	Prospective birth cohort study (CHEF)	559 children with PFAS serum measurements	PFAS levels in maternal serum (prenatal)	Asthma prevalence at ages five and 13	Prenatal PFAS exposure findings: no significant association was observed between prenatal PFAS exposure and asthma outcomes at ages five or 13, regardless of MMR vaccination status. This suggests that PFAS exposure during pregnancy may not directly impact asthma risk in early childhood	5/9
Atagi et al. [22]	2024	Japan	Prospective birth cohort study	17,856 mother-child pairs	PFAS levels in maternal serum (1st trimester); asthma symptoms in children at age four (ISAAC questionnaire)	"Wheeze ever", "asthma ever", doctor-diagnosed asthma	Initial associations: negative associations between PFOA and PFHxS levels with "wheeze ever" and "asthma ever". Final analysis: after Bonferroni correction, these associations were no longer statistically significant, indicating no conclusive link between prenatal PFAS exposure and asthma outcomes at age four	7/9
Zell-Baran et al. [23]	2023	USA	Prospective cohort study (Healthy Start cohort)	597 mother-child pairs	PFAS levels in maternal serum (mid-pregnancy); clinician-diagnosed asthma incidence through age eight	Asthma incidence and age at diagnosis	Asthma incidence: 17% of children diagnosed with asthma; median age at diagnosis: 1.7 years. Associations with PFAS exposure: elevated HRs for individual PFAS compounds and PFAS mixture; however, none were statistically significant, as all CIs included the null	7/9
Zeng et al. [24]	2024	China	Prospective birth cohort study (Shanghai Allergy Birth Cohort)	1,203 mother-child pairs	PFAS levels in cord blood; asthma and wheezing up to age five	Asthma and wheezing outcomes	Asthma prevalence: 17.4% of children were diagnosed with asthma by age five. PFAS exposure: no significant association between prenatal PFAS exposure and asthma or wheezing. IgE levels: positive correlation between cord serum PFAS levels (PFOA, PFOS, PFDA) and total serum IgE levels, particularly when exposure was above certain thresholds	7/9
Impinen et al. [25]	2019	Norway	Prospective birth cohort study (MoBa)	1,943 mother-child pairs from the Norwegian MoBa cohort	PFAS levels in maternal serum; asthma, atopic eczema, and infections in childhood up to age 7	Asthma and atopic eczema outcomes	PFUnDA and asthma: statistically significant inverse association between PFUnDA and asthma outcomes before FDR correction. Atopic eczema: inverse association between PFUnDA and atopic eczema, with a more pronounced effect in girls	7/9
Smit et al. [26]	2015	Greenland and Ukraine	Prospective birth cohort study	1,024 mother-child pairs from Greenland and Ukraine	PFAS levels in maternal serum; asthma and eczema outcomes at school age	Asthma and eczema prevalence	Asthma prevalence: in Greenland, 12.4% of children had asthma compared to 1.6% in Ukraine. PFOA was inversely associated with current wheezing in Ukrainian children (OR: 0.64, 95% CI: 0.41-0.99). Eczema prevalence: inverse association between organochlorines (PCBs and DDE) and ever eczema in Greenland (OR: 0.78, 95% CI: 0.61-0.99). Limited evidence: overall limited evidence linking prenatal exposure to environmental contaminants with asthma or eczema outcomes	7/9
Manzano-Salgado et al. [27]	2019	Spain	Prospective birth cohort study	1,108 children from a Spanish birth cohort	PFAS levels in maternal serum; asthma, eczema, and lung function outcomes up to age seven	Asthma outcomes	Prenatal PFNA exposure was associated with a reduced risk of asthma (RR: 0.74, 95% CI: 0.57-0.96)	7/9

Granum et al. examined the effects of prenatal exposure to PFAS, specifically perfluorooctanoate (PFOA), perfluorononanoate (PFNA), perfluorohexane sulfonate (PFHxS), and perfluorooctane sulfonate (PFOS), on immune outcomes in early childhood. No significant associations were found between PFAS exposure and doctor-diagnosed asthma or wheezing by age three. However, elevated PFAS levels were linked to reduced anti-rubella antibody levels, suggesting compromised vaccine response. Additionally, increased PFAS exposure, particularly to PFOA, PFNA, and PFHxS, was associated with a higher frequency of common colds and gastroenteritis episodes, indicating possible immunosuppressive effects of prenatal PFAS exposure on early childhood immune function [13].

Impinen et al. assessed the impact of prenatal exposure to six PFAS compounds on respiratory and allergic outcomes in children up to age 10. Although no significant associations were found between prenatal PFAS levels and asthma, wheezing, allergic rhinitis, or atopic dermatitis (a type of eczema), certain PFAS compounds were linked to increased respiratory infections. Specifically, PFOS, PFOA, and perfluoroundecanoic acid (PFUnDA) were associated with higher rates of respiratory tract infections, including lower respiratory infections, in the first 10 years of life, suggesting possible immunosuppressive effects of PFAS exposure on childhood infection susceptibility [14].

Beck et al. [15] evaluated the associations between prenatal exposure to five PFAS compounds [PFOS, PFOA, PFHxS, PFNA, and perfluorodecanoic acid (PFDA)] and asthma outcomes in 981 five-year-old children from the Odense Child Cohort. The findings showed no significant associations between prenatal PFAS levels and doctor-diagnosed asthma or wheezing. However, higher prenatal exposure to PFNA was significantly associated with an increased likelihood of self-reported asthma [odds ratio (OR): 1.84, 95% confidence interval (CI): 1.03-3.23]. Sex-specific analysis suggested that girls might have a higher risk of doctor-diagnosed asthma with elevated PFHxS exposure, though sample sizes were limited for these stratifications.

Philippat et al. [18] assessed prenatal exposure to multiple PFAS compounds and their effects on respiratory health outcomes in children up to age three within the SEPAGES cohort of 433 mother-child pairs. Although moderate PFAS exposure was associated with better lung function metrics (higher reactance and lower resistance at 36 months), no significant associations were found between PFAS exposure and asthma, wheezing, or other respiratory diseases in early childhood. Cluster analysis did not reveal any detrimental effects of PFAS on respiratory health outcomes, and individual PFAS compounds showed no consistent associations with respiratory disease or asthma indicators.

Begum et al. [19], in their study conducted within the Vitamin D Antenatal Asthma Reduction Trial (VDAART), examined the association between prenatal PFAS exposure and respiratory outcomes in children. The study involved 459 mother-child pairs and measured nine distinct PFAS compounds in maternal plasma samples collected in the third trimester. Results showed that while adjusted hazard ratios (HRs) for asthma were elevated for some PFAS compounds, none reached statistical significance after adjustments, and no consistent associations were observed between prenatal PFAS levels and childhood asthma incidence. This suggests no conclusive link between prenatal PFAS exposure and an increased risk of asthma in early childhood.

Sevelsted et al. [20], in their study as part of the Copenhagen Prospective Studies on Asthma in Childhood (COPSAC2010) cohort, analyzed prenatal and early childhood PFOS and PFOA exposure in relation to asthma phenotypes at age six among 738 children. Higher maternal levels of PFOS and PFOA during pregnancy were associated with an increased risk of non-atopic asthma (asthma not associated with allergies) but showed no significant association with atopic asthma (allergic asthma), lung function, or atopic dermatitis. Prenatal PFAS exposure appeared specifically linked to non-atopic asthma, with no impact from postnatal exposure. The study did not find any PFAS exposure effects on infection rates, systemic inflammation, immune response, or epigenetic markers, supporting a potential in-utero programming effect limited to non-atopic asthma.

Timmermann et al. [21] examined PFAS exposure at three different time points: prenatal (maternal serum), and postnatal at ages 5 and 13 years - and its association with asthma and allergies, considering the effect of measles, mumps, and rubella (MMR) vaccination status. Among 559 children, the results showed that PFAS exposure at age five was associated with an increased risk of asthma in MMR-unvaccinated children, while MMR-vaccinated children displayed an inverse trend. No association was found between prenatal PFAS exposure and asthma or allergic outcomes. These findings suggest that MMR vaccination may modify the effect of PFAS exposure on asthma risk.

Atagi et al. [22] explored the relationship between prenatal exposure to PFAS and respiratory outcomes in 17,856 mother-child pairs from the Japan Environment and Children’s Study. Maternal serum concentrations of six PFAS compounds were assessed during the first trimester. The findings indicated an association between higher PFOA levels and reduced occurrences of “wheeze ever” and “asthma ever,” with adjusted ORs suggesting a mild protective effect; however, these associations were no longer statistically significant after applying Bonferroni correction. Overall, no clear relationship between prenatal PFAS exposure and the prevalence of wheezing or asthma symptoms in four-year-old children was observed.

Zell-Baran et al. [23] examined 597 mother-child pairs to evaluate the association between maternal serum PFAS concentrations during pregnancy and the incidence of clinician-diagnosed asthma up to age eight. The study found that although adjusted HRs for asthma were elevated across individual PFAS compounds (PFHxS, PFOS, PFOA, PFNA, and PFDA), none reached statistical significance as all 95% confidence intervals included the null. A mixture analysis of the five PFAS compounds also showed no significant association with asthma risk, with a one-quartile increase yielding an HR of 1.17. This study highlights no consistent link between prenatal PFAS exposure and early childhood asthma.

Zeng et al. [24] conducted a study on prenatal exposure to eight PFAS compounds and their association with asthma and wheezing in preschool children as part of the Shanghai Allergy Birth Cohort. The study included 358 children, and the asthma diagnosis was made by pediatric respiratory physicians based on clinical assessments, including wheezing and immunoglobulin E (IgE) tests. The results revealed no significant association between prenatal PFAS exposure and the incidence of asthma or wheezing. However, certain PFAS compounds, such as PFOA, PFOS, and PFDA, were positively correlated with higher total IgE levels in five-year-old children at concentrations beyond a specific threshold, suggesting potential immunomodulatory effects. These findings suggest that while PFAS exposure may influence immune responses, no direct link was found between prenatal PFAS exposure and asthma development in this cohort.

Impinen et al. [25] explored the associations between prenatal PFAS exposure and asthma-related outcomes in the Norwegian Mother and Child Cohort (MoBa). The study included six PFAS compounds measured in maternal plasma and examined their effects on doctor-diagnosed asthma and parent-reported symptoms at age seven. Results indicated that prenatal PFAS exposure, particularly to perfluoroundecanoic acid (PFUnDA), was inversely associated with asthma, suggesting a potential protective or immunosuppressive effect. However, no significant associations were found between other PFAS compounds and asthma after controlling multiple comparisons. These findings align with previous studies, showing inconsistent results regarding the role of PFAS in childhood asthma development.

Smit et al. [26] conducted a study to explore the associations between prenatal exposure to a range of environmental contaminants, including PFAS, and the incidence of asthma and eczema in school-age children from the INUENDO birth cohort in Greenland and Ukraine. The study included 1024 mother-child pairs and applied principal component analysis (PCA) to assess the impact of multiple contaminants. The results showed limited evidence of a link between prenatal PFAS exposure and asthma. Specifically, the analysis revealed no consistent associations between PFAS exposure and asthma symptoms across the two populations.

Manzano-Salgado et al. [27] investigated prenatal exposure to four PFAS compounds and their associations with immune-related outcomes and lung function in children up to age seven in the Spanish INMA birth cohort study. Among 1,188 children, the study found an inverse association between PFNA exposure and asthma, suggesting that higher prenatal PFNA levels were associated with a reduced risk of asthma in childhood. Similarly, PFOS exposure was linked to a lower risk of eczema. However, prenatal exposure to PFOA was associated with reduced lung function at age four, indicated by lower forced vital capacity (FVC) and forced expiratory volume in 1 second (FEV1). These findings suggest that different PFAS compounds may have varying effects on respiratory and immune health, with some potentially lowering the risk of asthma but others impairing lung development.

Subgroup Analysis by Region

We conducted a subgroup analysis based on the geographic location of each study. Studies were categorized by region, covering Europe (Norway, Denmark, France, Spain, Greenland, and Ukraine), North America (USA), and Asia (Japan and China).

Despite diverse environmental exposures and varying background levels of PFAS, no consistent regional pattern emerged in the association between prenatal PFAS exposure and childhood asthma outcomes. Most studies, regardless of region, reported no significant association between prenatal PFAS exposure and doctor-diagnosed asthma or wheezing in children. This consistency across regions suggests that the observed lack of association is robust to geographic variability in PFAS exposure. Nevertheless, this finding underscores the need for future research to continue examining regional variations in PFAS exposure to clarify any localized impacts that may arise from differences in industrial or environmental PFAS levels.

Sensitivity Analysis by PFAS Compound and Exposure Levels

To address the potential impact of varying PFAS compounds and exposure levels, we examined studies based on the specific PFAS compounds assessed, primarily focusing on PFOA, PFOS, PFNA, and PFHxS. While individual studies examined different PFAS compounds, no single compound consistently demonstrated a significant association with asthma outcomes across studies. For instance, Beck et al. [15] found a significant association between self-reported asthma and PFNA exposure, but similar associations were not observed in other studies measuring PFNA. Similarly, Sevelsted et al. [20] reported increased odds of non-atopic asthma with higher prenatal PFOS and PFOA exposure, while studies from other regions reported null results for these compounds.

Additionally, studies that stratified participants by exposure levels (such as quartiles) did not observe significant trends linking higher PFAS exposure with asthma outcomes, as seen in Begum et al. [19]. The lack of consistent findings across both compound types and exposure levels indicates that no specific PFAS compound or threshold appears uniquely associated with increased asthma risk. This suggests that the overall impact of PFAS on asthma outcomes may be minimal or variable across different populations and PFAS types.

Sensitivity Analysis by Study Quality (NOS Scores)

Given the diversity in study quality, we further analyzed findings based on the NOS scores assigned to each study. Studies with higher NOS scores (7/9 or above) generally showed no association between prenatal PFAS exposure and asthma, providing a more consistent pattern of null results. For instance, Philippat et al. [18] and Atagi et al. [22], both scoring 7/9, reported no significant associations between prenatal PFAS exposure and asthma outcomes.

In contrast, lower-quality studies with NOS scores below 7 exhibited a mix of findings, with some reporting slight associations between certain PFAS compounds and asthma, such as Granum et al. [13]. However, these associations were limited and inconsistent across studies. Therefore, studies of higher methodological quality corroborate a null association between prenatal PFAS exposure and childhood asthma, reinforcing the reliability of our conclusion that no significant relationship exists in the overall analysis.

Discussion

This systematic review of 13 studies examined the association between prenatal exposure to PFAS and asthma in children. Despite variations in sample sizes, countries, and PFAS compounds measured, the studies generally found no consistent association between prenatal PFAS exposure and doctor-diagnosed asthma or wheezing in early childhood. These findings are in line with a previous systematic review focused on allergic outcomes in childhood which showed no significant association between PFOS, PFOA, PFHxS, or PFNA and childhood asthma, including prenatal exposure to these chemicals [28].

In contrast, a cross-sectional study has found that current PFOA levels in children were associated with higher odds of asthma in 12-19-year-olds [29]. Although this study did not incorporate prenatal measurements, it demonstrates that PFAS exposure should be evaluated at different time points of development [29]

PFAS contribute to asthma pathophysiology through their impact on airway inflammation, surfactant function, and immune modulation. PFAS, including PFOA and PFOS, are known to accumulate in lung tissues, disrupting the normal functions of lung surfactants and activating inflammatory pathways. For instance, studies have shown that PFOA can induce the activation of the NLRP3 inflammasome in human bronchial epithelial cells, which triggers the release of pro-inflammatory cytokines like interleukin- 6 (IL-6) and IL-1β, contributing to airway inflammation and asthma exacerbation​ [30, 31]. Moreover, PFOA alters membrane plasticity, impacting cellular structure, and may promote epithelial-to-mesenchymal transition (EMT), further aggravating lung pathology​ [30].

Chronic exposure to PFAS during critical developmental periods is hypothesized to enhance susceptibility to asthma by increasing airway hyperresponsiveness (AHR) and inflammation. Animal models have shown that prenatal and postnatal exposure to PFAS leads to increased airway resistance and a rise in lung macrophage populations, which can perpetuate the inflammatory response​ [32]. Notably, PFOA exposure has been associated with exacerbating T-helper 2 (Th2)-type airway inflammation and reducing the expression of the glucocorticoid receptor (GR), which may contribute to decreased responsiveness to asthma treatments like glucocorticoids​ [33]. Together, these findings indicate that PFAS exposure influences multiple mechanistic pathways involved in asthma, from surfactant dysfunction to immune dysregulation, potentially heightening asthma risk and severity.

Although strong mechanistic evidence suggests that PFAS, particularly compounds like PFOA and PFOS, contribute to asthma development by inducing airway inflammation, surfactant dysfunction, and immune dysregulation, the findings of this systematic review paint a more inconsistent picture. Experimental studies have demonstrated that PFAS activate inflammatory pathways, disrupt lung surfactant function, and skew immune responses toward an allergic phenotype, which could increase asthma risk [32]. However, the epidemiological studies in the review found no consistent association between prenatal PFAS exposure and childhood asthma, with several reporting null or mixed results. Some even suggested neutral or potentially protective effects for certain PFAS compounds [27], contradicting the experimental data. These discrepancies may stem from differences in study design, the types of PFAS examined, the timing of exposure measurements, and varying outcome definitions. This contrast highlights the need for more research to bridge the gap between mechanistic insights and real-world epidemiological evidence, especially in understanding under what conditions PFAS exposure may heighten asthma risk.

The comprehensive systematic review followed PRISMA guidelines, ensuring a systematic and rigorous approach to identifying relevant studies across multiple databases. This adherence to established protocols adds robustness to the findings and enhances the transparency of the study selection process. The inclusion of studies from diverse populations, spanning countries such as Norway, the USA, China, and Japan, provides a broad and globally representative data set, which increases the generalizability of the findings across various populations and settings. Additionally, the use of the NOS to assess the methodological quality of included studies ensures that only those with a high level of evidence and minimal bias were included, which strengthens the reliability of the review’s conclusions. Focusing specifically on prenatal exposure to PFAS offers valuable insights into how these environmental factors might impact respiratory health during critical developmental windows, contributing to a deeper understanding of asthma risk factors in early life.

One of the major limitations of the study is the heterogeneity in study designs, with included studies varying widely in terms of sample sizes, follow-up duration, and population characteristics. This heterogeneity limits the ability to synthesize the results in a coherent manner and may account for some of the inconsistencies in the findings. A significant limitation of this review is the variability in PFAS exposure assessment methods across studies. PFAS levels were measured in different biological samples, such as maternal blood, cord blood, and varying pregnancy stages, each with distinct half-lives and exposure windows that could influence exposure quantification. Additionally, studies examined different PFAS compounds, potentially resulting in inconsistent findings due to varied compound-specific effects on respiratory health. These methodological differences hinder direct comparisons between studies and may contribute to the mixed findings reported in this review. 

Furthermore, while many studies controlled for confounding variables, residual confounding from unmeasured factors, such as environmental or genetic variables, may have still influenced the observed associations. Asthma development likely results from interactions between genetic predispositions and environmental exposures, both of which could confound the association between prenatal PFAS exposure and childhood asthma. Genetic factors, such as immune-related gene variations, may heighten susceptibility to asthma, while environmental factors like air pollution, allergens, and secondhand smoke are known to increase asthma risk [4, 34]. Additionally, the presence of chemical mixtures-PFAS alongside other persistent contaminants-may compound health impacts, making it crucial for future research to consider genetic profiles and broader environmental exposures to better clarify PFAS’s role in asthma pathogenesis [35]

The small number of studies included in the review, despite the diversity of populations covered, may not provide sufficient data to fully elucidate the complex relationship between prenatal PFAS exposure and asthma risk. Lastly, the inconsistency in how asthma and respiratory outcomes were defined and measured across studies could have contributed to variations in results, further complicating the interpretation of the findings.

Future research on the relationship between prenatal PFAS exposure and childhood asthma should address the limitations identified in the current body of evidence. Standardizing the measurement of PFAS exposure, both in terms of biological samples and the timing of assessment during pregnancy, will be key for improving the comparability of studies. Additionally, there is a need for larger, more diverse cohort studies with longer follow-up periods to capture asthma outcomes throughout childhood and adolescence, as the effects of PFAS may not become evident until later in development. It is also essential to conduct more studies that account for a broader range of confounding factors, including genetic predispositions and postnatal environmental influences, to provide a clearer understanding of how PFAS interact with other risk factors for asthma. Mechanistic studies should continue to explore the biological pathways through which PFAS may influence respiratory health, ideally in tandem with epidemiological studies, to bridge the gap between experimental findings and real-world outcomes. Finally, a focus on vulnerable subpopulations, such as those with pre-existing health conditions or those living in areas with higher PFAS exposure, could offer more targeted insights into the potential risks associated with prenatal PFAS exposure.

## Conclusions

This systematic review highlights the inconsistent findings regarding the association between prenatal PFAS exposure and childhood asthma. While mechanistic studies suggest that PFAS contribute to asthma pathophysiology through airway inflammation, surfactant disruption, and immune modulation, the epidemiological evidence remains inconclusive. Most studies have reported null or mixed findings, with a few suggesting potential protective effects of certain PFAS compounds. These discrepancies may be attributed to differences in study design, exposure measurements, and confounder control. Future research should aim for standardized exposure assessment, preferably through larger cohort studies, and a closer alignment between mechanistic and epidemiological data to clarify the impact of PFAS on childhood asthma.
